# Tuning magnetotransport in a compensated semimetal at the atomic scale

**DOI:** 10.1038/ncomms9892

**Published:** 2015-11-24

**Authors:** Lin Wang, Ignacio Gutiérrez-Lezama, Céline Barreteau, Nicolas Ubrig, Enrico Giannini, Alberto F. Morpurgo

**Affiliations:** 1 Department of Quantum Matter Physics, Universite de Geneva, 24 quai Ernest Ansermet, CH-1211 Geneva, Switzerland; 2 Group of Applied Physics, Universite de Geneva, 24 quai Ernest Ansermet, CH-1211 Geneva, Switzerland

## Abstract

Either in bulk form, or in atomically thin crystals, layered transition metal dichalcogenides continuously reveal new phenomena. The latest example is 1T'-WTe_2_, a semimetal found to exhibit the largest known magnetoresistance in the bulk, and predicted to become a topological insulator in strained monolayers. Here we show that reducing the thickness through exfoliation enables the electronic properties of WTe_2_ to be tuned, which allows us to identify the mechanisms responsible for the observed magnetotransport down to the atomic scale. The longitudinal resistance and the unconventional magnetic field dependence of the Hall resistance are reproduced quantitatively by a classical two-band model for crystals as thin as six monolayers, whereas a crossover to an Anderson insulator occurs for thinner crystals. Besides establishing the origin of the magnetoresistance of WTe_2_, our results represent a complete validation of the classical theory for two-band electron-hole transport, and indicate that atomically thin WTe_2_ layers remain gapless semimetals.

## Abstract (web summary)

Semimetallic WTe_2_ possesses very high magnetoresistance however conflicting pictures of the band structure responsible have emerged. Here, the authors find behaviour consistent with a model of two compensated electron and hold bands in exfoliated WTe_2_ films with thicknesses down to the atomic scale.

## Graphical abstract

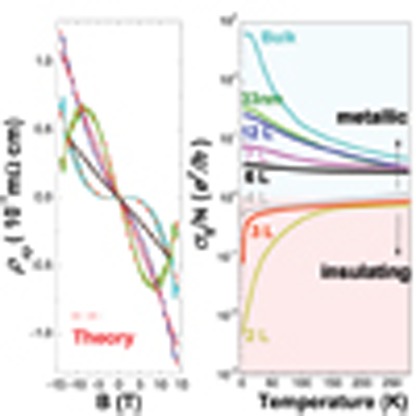

Semimetallic compounds are known to often exhibit many unusual electronic properties, including a non-saturating magnetoresistance (MR) with values among the largest ever reported^1^,^2^,^3^,^4^,^5^,^6^,^7^,^8^. Different theoretical scenarios—based on charge inhomogeneity^1^,^3^, spin–orbit interaction^5^, or the linear dispersion relation of charge carriers^2^,^5^,^7^,^8^ have been proposed in the past to explain such a large MR, but finding experimental evidence to conclusively establish their relevance for experiments has proven extremely difficult. The problem has resurfaced recently with the discovery of a record-high MR in semimetallic^9^,^10^ bulk 1T'-WTe_2_ (ref. 6), which was suggested to originate from the classical magnetotransport properties of two, nearly perfectly compensated electron and hole bands. The results of even more recent quantum oscillations^11^,^12^,^13^,^14^ and angle resolved photoemission (ARPES)^15^,^16^ measurements, however, suggest that more than two bands are present at the Fermi level, and cast doubts about the validity of this conclusion.

To address this issue, we have investigated magnetotransport through exfoliated WTe_2_ crystals and explored the properties of this material from bulk crystals all the way to atomically thin layers. We find that in all cases—for thicknesses down to six monolayers—the measured longitudinal and transverse MR can be reproduced with remarkable accuracy by a classical two-band model describing simultaneous electron and hole transport. For smaller thicknesses, the transport properties of WTe_2_ crystals change, and for crystals that are four monolayer or thinner we observe a transition to an insulating state. This insulating state occurs concomitantly with a qualitative change in the magentotransport properties that become fully dominated by quantum interference effects. All the observed phenomenology indicates that the insulating state originates from Anderson localization caused by an enhanced scattering at the outermost (surface) layer of the material, degraded by exposure to air. This finding implies that even down to the ultimate atomic thickness the WTe_2_ crystals remain un-gapped semimetals.

## Results

### Magnetotransport of WTe_2_ in terms of a two-band model

According to theory^17^,^18^, classical magnetotransport in a two-band nearly compensated semimetal is described by the following expressions for the longitudinal and transverse resistivity *ρ*_*xx*_(*B*) and *ρ*_*xy*_(*B*) (where *n*, *p*, *μ*_e_ and *μ*_h_ are electron and hole densities and mobility and *B* is the magnetic field):


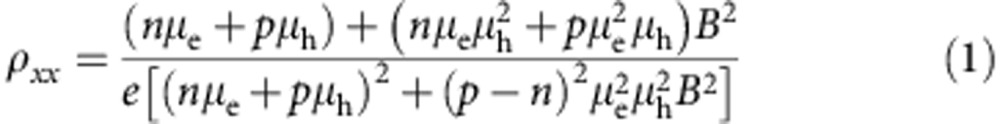



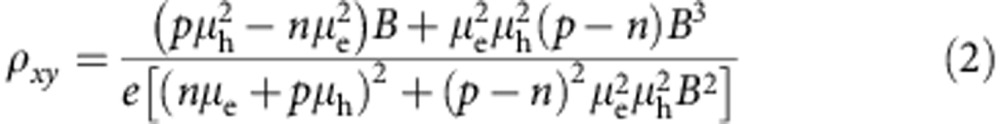


so that the MR is given by:


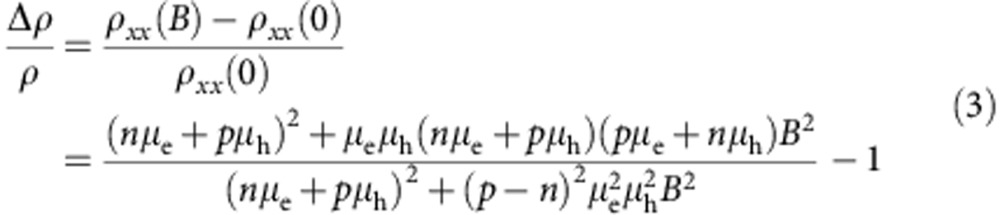


The regime of near compensation corresponds to having *n* sufficiently close to *p* so that the *B*^2^ term in the denominator of equations (1)–(3), 2, 3 can be neglected in the magnetic field range explored in the experiments (that is, 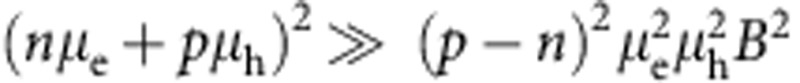 for all values of *B* in the measurements). In this case, the MR increases quadratically without saturation, 
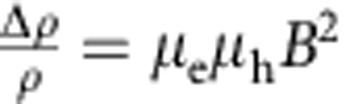
, which is why this scenario was originally invoked to explain the behaviour of WTe_2_ (ref. 6). What is peculiar in this regime is the behaviour of the Hall resistivity *ρ*_*xy*_(*B*): it is proportional to *B* at low field and to *B*^3^ at high field, with the magnitude and sign of the proportionality coefficients of both terms depending sensitively on the relative values of *n*, *p*, *μ*_e_ and *μ*_h_. For nearly compensated systems, small relative changes in *p* and *n* (or in *μ*_e_ and *μ*_h_) can then lead to dramatic changes in the behaviour of *ρ*_*xy*_(*B*), so that—if the values of the system parameters *n*, *p*, *μ*_e_ and *μ*_h_ can be tuned experimentally—monitoring the evolution of *ρ*_*xy*_(*B*) allows the validity of the proposed scenario to be proven (or disproven) unambiguously. However, virtually no effort has been devoted so far to investigating *ρ*_*xy*_(*B*) on changing the system parameters, largely because of the experimental difficulties involved in controlling and determining unambiguously *p*, *n*, *μ*_e_ and *μ*_h_.

**Figure 1:**
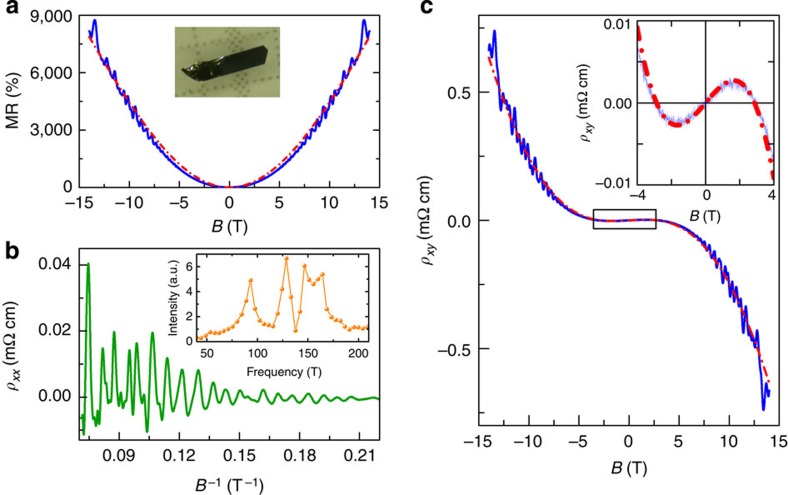
Magnetotransport of bulk WTe_2_. (**a**) Longitudinal MR of bulk WTe_2_ showing a non-saturating, quadratic field dependence—as expected from the classical theory for a compensated semimetal (here and in panel **c**, the blue line represents the experimental data and the red dashed-dotted line the theoretical fit with equations (2) and (3), respectively). SdH oscillations are also clearly visible. The inset shows an optical image of a WTe_2_ crystal (the scale of the graded paper is 1 × 1 mm). (**b**) SdH oscillations obtained by subtracting the quadratic background from the longitudinal resistivity, plotted as a function of *B*^*−*1^. The dominant peaks in the frequency spectrum, shown in the inset, occur at 93, 129, 147 and 164 T, in virtually perfect agreement with very recent work^11^,^12^,^13^,^14^. (**c**) The Hall resistivity, *ρ*_*xy*_, exhibits a very unconventional behaviour: it is linear at low *B* (see inset), proportional to *B*^3^ at high fields, and changes sign at ∼B*=±3 T. This rich behaviour—that had never been reported previously for any semimetal—is perfectly captured, at a quantitative level, by equation (2) in the main text (represented by the red dashed-dotted line). All measurements shown here have been performed at *T=*250 mK.

WTe_2_ is rather unique in this regard because the material is layered (see Supplementary Fig. 1 for the crystal structure), and the system parameters can be varied by changing the material thickness through a simple exfoliation process. In addition, for WTe_2_ the comparison between experiment and theory is facilitated by the possibility to extract accurate estimates for the parameters directly from the experiments, which drastically narrows down the parameter range when fitting equations (1)–(3), 2, 3 to the data. We start with the analysis of magnetotransport of bulk crystals. Figure 1a,b shows—as reported in recent earlier studies^6^,^11^,^12^,^13^,^14^—that the relative MR exhibits a large, non-saturating quadratic dependence on *B*, and that Shubnikov–de Haas (SdH) oscillations are clearly visible (for *B*≳3 T). The oscillation spectrum exhibits four independent frequencies ranging from ∼90 to 170 T, whose attribution to different families of charge carriers has not yet been conclusively established^11^,^12^,^13^,^14^. We follow ref. 12 and assume that the Fermi surface is approximately ellipsoidal with a circular cross-section in the plane perpendicular to *B*, which enables us to extract the value of the Fermi momentum *k*_F_ and estimate the density of carriers (whose value depends on the assumed degree of anisotropy of the ellipsoidal Fermi surface; see ref. 12 for details). This estimate serves to fix the starting values of *n* and *p* that we use in fitting the data with equations (1)–(3), 2, 3. From the analysis of the SdH oscillations we also extract fairly precise estimates for *μ*_e_ and *μ*_h_: the value of *B* at which the SdH oscillations appear fixes the mobility of one type of charge carriers through the condition *μB*∼1; the mobility for the other carrier type can then be estimated from the magnitude of the quadratic MR, 
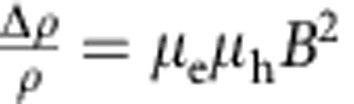
 (determining which type of charge carriers has one or the other mobility value requires the full data analysis).

Starting from the estimated values of *n*, *p*, *μ*_e_ and *μ*_h_, we perform a fully quantitative fit of the experimental data to equations (1)–(3), 2, 3. The outcome of the fitting procedure is represented in Fig. 1a,c (see also the inset) with red dashed-dotted lines, for both *ρ*_*xx*_(*B*) and *ρ*_*xy*_(*B*). We obtain an excellent agreement between measurements and theory with values of parameters that are very close to our initial ‘rough' estimates (for the electron and hole density, the final values are typically within a factor of two of the initial guesses or better, except for very thin layers, in which the SdH oscillations are weak; for the mobility values the deviation between initial guesses and final values is typically around 30%). The agreement between theory and data is particularly remarkable and compelling for *ρ*_*xy*_(*B*): this quantity is ‘independent' of the measurements used for the initial estimate of the model parameters (that is, *ρ*_*xy*_(*B*) was not used to estimate the values of the parameters), and yet the analysis fully reproduces at a qualitative and quantitative level the extremely unconventional behaviour observed experimentally. As shown in the inset of Fig. 1c, theory even reproduces correctly the change in sign of *ρ*_*xy*_(*B*) as a function of *B*, whose observation has never been previously reported in a semimetal. As equation (2) predicts the zero crossings to occur at 
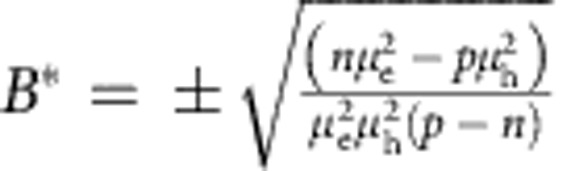
, this observation imposes a tight quantitative constraint on the model parameters. It is the unique nature of the behaviour observed experimentally, together with the excellent agreement between data and equations (1)–(3), 2, 3, that allow us to conclude unambiguously that a nearly compensated two-band model in the classical regime explains the magnetotransport properties of WTe_2_.

### Thickness evolution of classical magnetotransport

**Figure 2:**
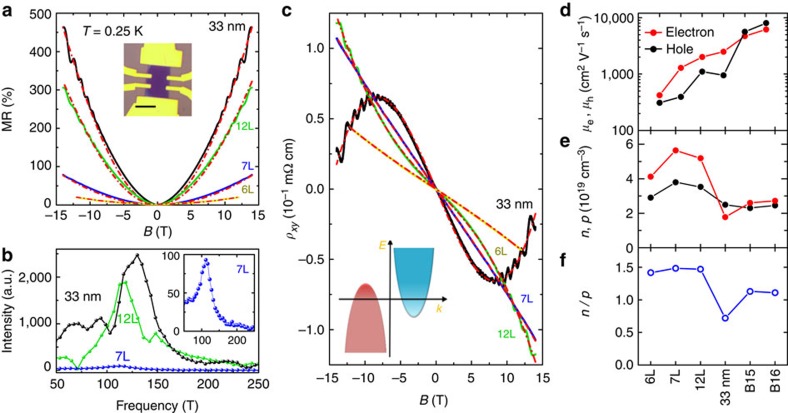
Evolution of magnetotransport in WTe_2_ crystals from bulk to the atomic scale. (**a**) The continuous lines represent the longitudinal MR measured in exfoliated crystals of different thickness (black, 33 nm; green, 12 layers −12L; blue; 7 layers −7L; dark yellow, 6 layers −6L). The red dashed-dotted lines are theoretical fits to equation (3) in the main text. Just as for the bulk, the MR does not saturate, is quadratic throughout the experimental range, and is perfectly reproduced by theory. The inset shows an optical image of a 7L device (scale bar, 5 μm). SdH oscillations superimposed on the measurements of all devices are also visible down to a thickness of 7L, starting from increasingly larger magnetic field values. The corresponding spectra of the oscillations are shown in panel **b** and its inset; at small thicknesses the smaller number of periods visible in the oscillations decreases the frequency resolution, limiting the visible peak substructure. (**c**) Transverse resistivity *ρ*_*xy*_ of the same devices for which the MR is shown in panel **a**. The different functional dependencies observed for different thicknesses are fully reproduced by equation (2) in the main text (red dashed-dotted lines; all measurements in this figure are done at *T=*250 mK). The inset shows a schematic representation of the low-energy electronic structure of a two-band semimetal. Panels **d**–**f** summarize the evolution of the electron (red) and hole (black) mobility, density and their ratio (density), respectively, as extracted from fitting the data to equations (2) and (3).

By following the procedure established for macroscopic crystals, we extend our investigations to layers of increasingly smaller thickness, which enables us to explore whether WTe_2_ behaves as a compensated two-band semimetal even when its thickness is reduced to the atomic scale^19^,^20^. Transport measurements were performed on devices nanofabricated on exfoliated flakes whose thickness was determined—all the way to individual monolayers—through a careful analysis relying on Raman spectroscopy^21^,^22^,^23^, optical contrast measurements and atomic force microscopy (see Supplementary Note 1 and Supplementary Figs 2–4). Figure 2a shows the MR of exfoliated crystals with thickness down to six monolayers, together with the results (red dashed-dotted lines) of the quantitative fitting to equations (1)–(3), 2, 3. In all cases, we find that theory fully reproduces all quantitative and qualitative aspects of the data for both *ρ*_*xx*_(*B*) and *ρ*_*xy*_(*B*) (see Fig. 2a,c; clearly, the SdH oscillations that are superimposed on the classical longitudinal and transverse resistivity are of quantum origin and are therefore not reproduced by the classical theory). On varying the thickness, the longitudinal MR always exhibits a *B*^2^ dependence, whereas the functional dependence of *ρ*_*xy*_(*B*) varies very considerably (presence or absence of non-monotonicity, strength of the non-linearity, concavity/convexity of the curve and so on.). This rich behaviour—very precisely reproduced by equation (2)—is a manifestation of the changes in sign (and magnitude) of the coefficients of the *B*-linear and *B*-cubic terms, determined by the relative magnitude of *n* and *p*, and of *μ*_e_ and *μ*_h_.

The very systematic agreement between data and equations (1)–(3), 2, 3 is quite remarkable in two important regards. Firstly, it represents a complete quantitative validation of the classical theory of transport for a nearly compensated semimetal with an electron and a hole band, at a notably high level of detail. Secondly, it shows that a two-band nearly compensated semimetal model does reproduce the magnetotransport properties of WTe_2_ very satisfactorily in a way that is insensitive to the precise details of the material band structure, which on the energy scale of the band overlap—few tens of meV—is certainly different for the bulk and for crystals that are only six or seven monolayer thick. This insensitivity to details is very likely the reason why a two-band model works so well, despite the presence of more bands, as clearly indicated from SdH oscillations and ARPES measurements. It strongly suggest that electrons (and holes) in different bands have essentially the same mobility, so that classical transport is only sensitive to their total density (and not to the density in each one of the bands). Indeed, if we compare the compensation level *n*/*p* that we extract from classical transport with values reported earlier, inferred from the total electron and hole density obtained from the SdH oscillations frequencies^12^,^13^ or ARPES^15^,^16^ measurements in bulk crystals, we find a satisfactory agreement.

As it is apparent from the quality of the agreement between equations (1)–(3), 2, 3 and the data, the ability to reproduce different qualitative features with a same functional dependence allows all parameters in the model to be extracted precisely. Figure 2d–f summarize the evolution of *n*, *p*, *μ*_e_ and *μ*_h_ with decreasing thickness, which allows the identification of several trends. Bulk crystals with thickness on the mm scale (that is, samples B15 and B16) exhibit only small sample-to-sample fluctuations in electron and hole density and mobility: both electron and hole mobility values are rather large (between 5,000 and 10,000 cm^2^ V^−1^ s^−1^) and compensation between electrons and holes is nearly perfect (*n*/*p*∼1.1). As the layers are thinned down *μ*_e_ and *μ*_h_ decrease, because the crystal thickness becomes smaller than the mean free path and scattering at the surface becomes relevant. Nevertheless, even for the thinnest layers analysed—only six or seven-layer thick—*μ*_e_ and *μ*_h_∼1,000 cm^2^ V^−1^ s^−1^. The electron and hole densities *n* and *p* exhibit reproducible variations as a function of thickness (the minimum in *n* seen in Fig. 2e, for instance, has been found in the other crystals measured, having thickness between 30 and 50 nm). The origin of these variations is likely the consequence of different physical phenomena, whose relevance depends on the thickness range considered (such as strain for crystals that are several tens of nanometres thick, and the effect of charge transfer from the surface for thinner layers). The net result is that the compensation level worsens for thinner layers and the data show that *n*/*p* ranges from ∼0.7 to 1.5 as the thickness is reduced from bulk crystals to crystals that are only seven-layer thick. The dependence of *ρ*_*xx*_(*B*) remains, nevertheless, quadratic throughout the magnetic field range of our measurements, implying that 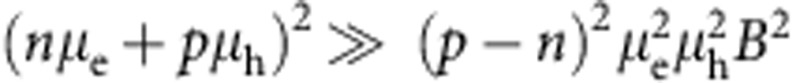, so that even thin layers still fall in the theoretical regime characteristic of nearly compensated semimetals. The magnitude of the MR is, however, very significantly suppressed, mainly because of the large drop in carrier mobility. We conclude that, although having comparable values for the density of electrons and holes is important, it is the high mobility of the two carriers that is essential to achieve the very large MR measured in WTe_2_.

### Metal-to-insulator transition in thin WTe_2_ flakes

**Figure 3:**
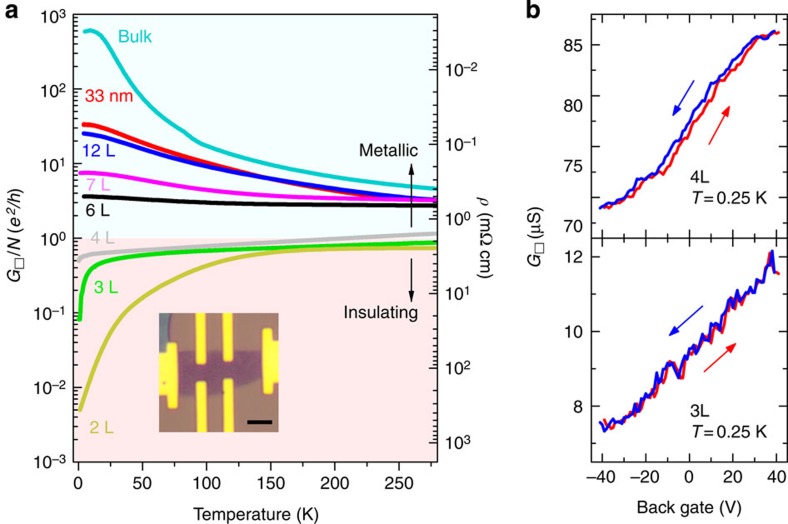
Metal–insulator transition in atomically thin WTe_2_. (**a**) Temperature dependence of the conductance per square normalized to the number of layers, *G*_*□*_/*N*, measured in crystals of different thickness (from bulk to bilayer −2L) as a function of temperature *T* (the right axis gives the corresponding resistivity values). The occurrence of a metal–insulator crossover is clearly apparent, with the six-layer (6L) device being still metallic and the four-layer one (4L) being the first for which insulating behaviour is observed. Note how the transition from metallic to insulator occurs at a conductivity per layer that is ∼*e*^*2*^/*h*. The inset is an optical image of a bilayer device (scale bar, 5 μm). (**b**) Square conductance, *G*_*□*_, of a trilayer (3L, top) and 4L (bottom) as a function of gate voltage, *V*_G_, measured at *T=*250 mK and *B=*0 T (the crystals are mounted on a highly doped silicon wafer acting as a gate, covered by a 285 nm SiO_2_ layer acting as gate insulator). The red and blue curves correspond to data taken on sweeping the gate voltage in opposite directions, as indicated by the arrows of the corresponding colour, and illustrate the reproducibility of the measurements.

As the thickness of WTe_2_ is decreased even further to approach the ultimate limit of individual monolayers, the transport regime of WTe_2_ changes qualitatively. The change manifests itself in a metal–insulator transition clearly visible in the temperature dependence of the conductivity, as shown in Fig. 3a. Interestingly, normalizing to the number of layers, we find that the transition occurs when the conductivity per layer is ∼*e*^*2*^/*h*. WTe_2_ crystals that are four-layer thick are on the insulating side of the transition—albeit just barely—and the insulating temperature dependence of the conductivity becomes progressively more pronounced for tri and bilayers (we have also fabricated monolayer devices and found that their conductance is unmeasurably low). In all cases, the observed insulating behaviour sets in only at relatively low temperature so that, whereas, the room temperature resistivity increases by only a factor of five when comparing bulk crystals and bilayers, the increase is as large as five orders of magnitude at *T*=250 mK.

Identifying the origin of the insulating state is important to fully understand the properties of WTe_2_ down to the ultimate atomic scale. For crystals only a few monolayers thick, changes in the band structure may reduce the overlap between conduction and valence bands, eventually leading to the opening of a band gap, a scenario that would account for the observed metal–insulator transition. Such an explanation, however, does not seem consistent with the experimental results. The analysis of magnetotransport, for instance, shows that the density of electrons and holes does not change significantly on thinning down the material. If anything, the electron density increases, whereas, a decrease in band overlap—and the opening of a small gap—should cause the opposite effect. In addition, the square conductance *G*_*□*_ measured in tri and four-layer WTe_2_ increases steadily with increasing gate voltage *V*_G_ (see Fig. 3b), showing no sign of the non-monotonic dependence expected for ambipolar conduction normally observed in the presence of a small band gap. On contrary, the *G*_*□*_(*V*_G_) dependence is consistent with the behaviour expected if both types of charge carriers are present, with electrons having a mobility 2-to-3 times larger than the holes (that is, the same behaviour seen in crystals that are 6-to-12 monolayer thick, see Fig. 2e). In this case, we can estimate *μ*_e_ for trilayers and four layers, by applying the relation 
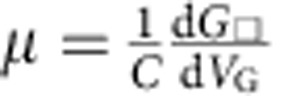
 (with *C* gate capacitance per unit area) to extend our estimates of carrier mobility; we find *μ*_e_∼25 cm^2^ V^−1^ s^−1^ and *μ*_e_∼5 cm^2^ V^−1^ s^−1^ in the two cases. These values, one-to-two orders of magnitude smaller than those found in the thinnest multilayers that still exhibit metallic behaviour (see Fig. 2e), indicate rather unambiguously that the insulating state originates from an increase in disorder strength. Indeed, our observation that the crossover from metallic to insulating behaviour occurs when the conductivity per layer is ∼*e*^*2*^/*h* strongly suggests that carriers in very thin WTe_2_ layers are Anderson localized.

### Quantum correction to the magnetoconductivity

**Figure 4:**
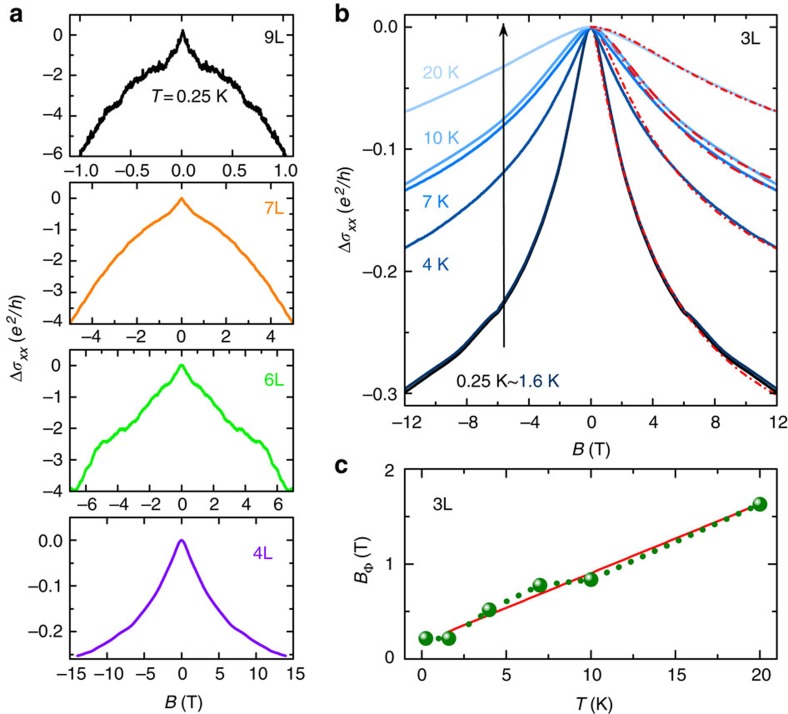
Quantum localization in atomically thin WTe_2_ crystals. (**a**) Longitudinal magnetoconductance of crystals of different thickness (from top to bottom, nine, seven, six and four layers, respectively), focusing on the magnetic field range where quantum interference effects are visible (data taken at *T=*250 mK). Quantum interference manifests itself in the WAL correction starting to be clearly visible in the 9L device; on decreasing the crystal thickness the relative magnitude of the effect of quantum interference increases. For the 4L device—the first exhibiting an insulating *T* dependence of the conductivity—quantum interference dominates magnetotransport, so that no quadratic MR of classical origin is visible. (**b**) Magnetic field dependence of the magnetoconductance of a trilayer device (for *T* ranging 250 mK–20 K), showing a decrease of the magnetoconductance with increasing temperature, as expected for quantum interference effects. The blue solid lines correspond to the experimental data; the red dashed lines represent theoretical curves obtained by fitting the data with the theory for WAL, equation (4). (**c**) Temperature dependence of *B*_φ_ extracted from fitting the trilayer magnetoconductance with equation (4). The linear temperature dependence of *B*_φ_ is consistent with dephasing caused by electron–electron interactions in a diffusive system. The red line is a guide to the eye.

To find additional experimental evidence supporting the tendency of charge carriers towards localization, we look at magnetotransport measurements of WTe_2_ crystals that are four layers or thinner. The emergence of quantum corrections to the conductivity in the low temperature MR data—superimposed on the quadratic classical background—becomes clearly apparent as the material thickness is decreased below 10 layers (see Fig. 4a). The phenomenon is known as weak antilocalization (WAL) and originates from the interference of quantum coherent electronic waves undergoing diffusive motion in the presence of spin–orbit interaction, which in WTe_2_ is large due to the large atomic number of tungsten. The relative strength of the effect increases as the layer thickness is decreased, because the magnitude of the WAL contribution on the conductivity is always ∼*e*^*2*^/*h*, whereas the classical contribution decreases on thinning down the material (to illustrate this point more clearly, in the Supplementary Fig. 5 the quantum correction signal is shown separately, after subtracting the classical contribution from the measured conductivity; see Supplementary Note 2 for more details). Note also that the magnetic field range in which WAL is visible becomes larger with decreasing thickness, as expected, because thinner flakes have smaller mobility and hence shorter electronic mean free path. Quantum interference effects eventually entirely dominate the MR of crystals thinner than four layers—the same thickness for which the insulating temperature dependence of the conductivity is first observed—that do not any more exhibit the quadratic *B* dependence expected from equations (1)–(3), 2, 3. We conclude that, in passing through the metal–insulator transition, the MR of WTe_2_ changes qualitatively, and its behaviour is determined by quantum—and not any more classical—processes. The absolute magnitude of the quantum magnetoconductance at *T*=250 mK is ∼*e*^*2*^/*h* and it decreases on increasing the temperature (see Fig. 4b), as qualitatively expected for the WAL correction to the conductivity.

Even though the precise nature of the spin–orbit interaction responsible for spin flip cannot be determined from the measurements, we attempt a semi-quantitative analysis of the data by fitting to Hikami–Larkin–Nagaoka theory for WAL, whose expression for the magnetoconductance reads^24^:


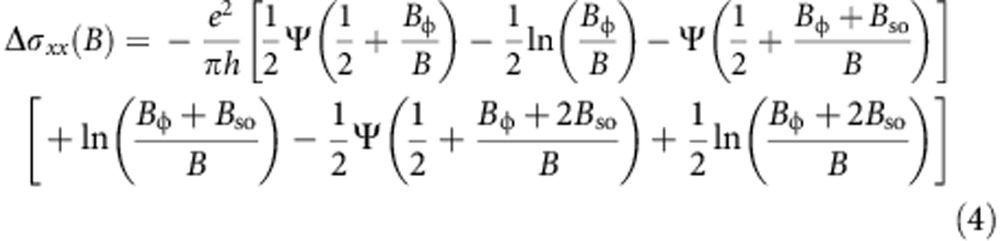


where Ψ is the digamma function, 
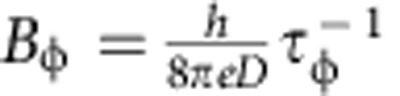
 is determined by the electron phase coherence time *τ*_φ_ and the diffusion constant *D*, and 
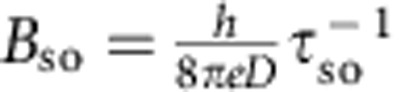
 is determined by the spin relaxation time *τ*_so_. Figure 4b shows that the agreement between measurements performed on trilayer WTe_2_ and theory is remarkably good. To fit the data we allow *B*_φ_ to vary as a function of temperature (as shown in Fig. 4c), and keep *B*_so_ constant (6 T), as expected from the physical meaning of these parameters (*B*_φ_ is proportional to the inverse of phase coherence time 
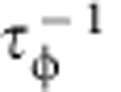
, and the approximately linear *T* dependence of *B*_φ_ is consistent with dephasing induced by electron–electron interactions in a diffusive system^25^). Irrespective of the precise values extracted for the parameters, which may depend on the specific theory of WAL used to fit, the agreement between equation (4) and the experimental data (as well as the internal consistency of the behaviour observed experimentally) confirm that in WTe_2_ crystals that are only a few layers thick localization effects dominate. Our results therefore show that—albeit localized—carriers are still present at the Fermi energy. This confirms the results of theoretical calculations^19^,^20^ that indicates how WTe_2_ remains as a gapless semimetal all the way down to monolayer thickness.

## Discussion

The increase in disorder that is responsible for the occurrence of Anderson localization in very thin WTe_2_ crystals originates from the non-perfect chemical stability of WTe_2_ in the presence of humidity. Such a non-perfect stability leads to a detectable change in colour and contrast (visible under an optical microscope, see Supplementary Note 3 and Supplementary Fig. 6) if thin crystals (<4 layers) are exposed to the environment for a sufficiently long time. It appears that between one and two layers on each crystal face are affected by the degradation process during the time needed to nano-fabricate devices (that includes optical inspection to identify the crystals, spinning and backing the resist needed for electron-beam lithography to define the metal contacts, and performing the lift off process after the metal deposition). As compared with many other exfoliated materials that react chemically when exposed to air—such as phosphorene^26^,^27^,^28^,^29^ and NbSe_2_ (ref. 30), for which strong degradation is visible after 1 h of exposure to air—WTe_2_ seems to be considerably more stable. Significant degradation, giving optically visible signs, is only seen to occur after exposure to the environment for a day or longer periods.

As it has been demonstrated for highly air-sensitive materials, encapsulation^27^,^28^,^29^ (for example, in between two hBN layers) of WTe_2_ layers under controlled atmosphere will allow material degradation during the device fabrication process to be eliminated. On the basis of the analysis of transport presented in this paper, we anticipate that preserving the chemical integrity of the crystal surface will prevent the decrease in carrier mobility and will enable the realization of atomically thin layers possessing carrier mobility values comparable to those found in the bulk. Such an advance will have important consequences. In particular, it will drastically increase the magnitude of the MR in atomic scale crystals of WTe_2_ to the record values observed in the bulk. It will also make WTe_2_ fully gate tunable: since mono, bi and trilayers are sufficiently thin to vary their carrier density significantly with electrostatic gate electrodes, the realization of gated encapsulated devices will disclose the possibility to perform experiments that are impossible to perform in thicker, bulk-like crystals. Finally, we envision that it will be possible to induce and control strain in encapsulated monolayer WTe_2_ using techniques similar to those employed in graphene^31^. This will open the possibility to perform transport experiments to test whether strained monolayers of 1T'-WTe_2_ are two-dimensional topological insulators as recently predicted theoretically^19^.

## Methods

### WTe_2_ crystal growth

All transport measurements described in this paper have been performed on devices based on WTe_2_ crystals grown by means of chemical vapour transport using WCl_6_ as a transport agent, as discussed in more detail in the Supplementary Note 4.

### Device fabrication

For measurements on bulk samples, electrical contacts were made with silver epoxy directly on suitably chosen as-grown crystals. To investigate transport on thinner layers, flakes were exfoliated from bulk crystals using adhesive tape and transferred onto a Si substrate covered with 285 nm of SiO_2_, after which conventional nano-fabrication techniques (electron-beam lithography, metals evaporation and lift off) were employed to attach electrical contacts (consisting of Ti/Au bilayers, typically 10/70 nm thick). The thickness of the exfoliated crystals was identified by means of atomic force microscopy, optical contrast and Raman spectra as discussed in detail in the Supplementary Note 1. Throughout the process of crystal identification and device fabrication, care was taken to minimize exposure of the material to air to minimize degradation (the exfoliated crystals where stored in either a glove box with sub-p.p.m. concentration of oxygen and water, or in a high vacuum chamber, when not being processed).

### Measurements

All magnetotransport measurements were performed using either a Heliox ^3^He system (Oxford instruments) operated in a cryostat with a 14 T magnet and a base temperature of 250 mK, or a cryofree Teslatron cryostat with a 12 T magnet and a base temperature of 1.5 K. The measurements were performed in a current-bias configuration using lock-in amplifiers and home-made low-noise electronic current sources and voltage amplifiers.

## Additional information

**How to cite this article:** Wang, L. *et al.* Tuning magnetotransport in a compensated semimetal at the atomic scale. *Nat. Commun.* 6:8892 doi: 10.1038/ncomms9892 (2015).

## Supplementary Material

Supplementary Information Supplementary Figures 1-6, Supplementary Notes 1-4 and Supplementary References.
