# Supplementary material for: Tuning magnetotransport in a compensated semimetal at the atomic scale
Source: Nat Commun. 2015 Nov 24;6:8892. doi: 10.1038/ncomms9892 (PMC4673487; doi:10.1038/ncomms9892)
Supplement: Supplementary Information — Supplementary Figures 1-6, Supplementary Notes 1-4 and Supplementary References. [file ncomms9892-s1.pdf]

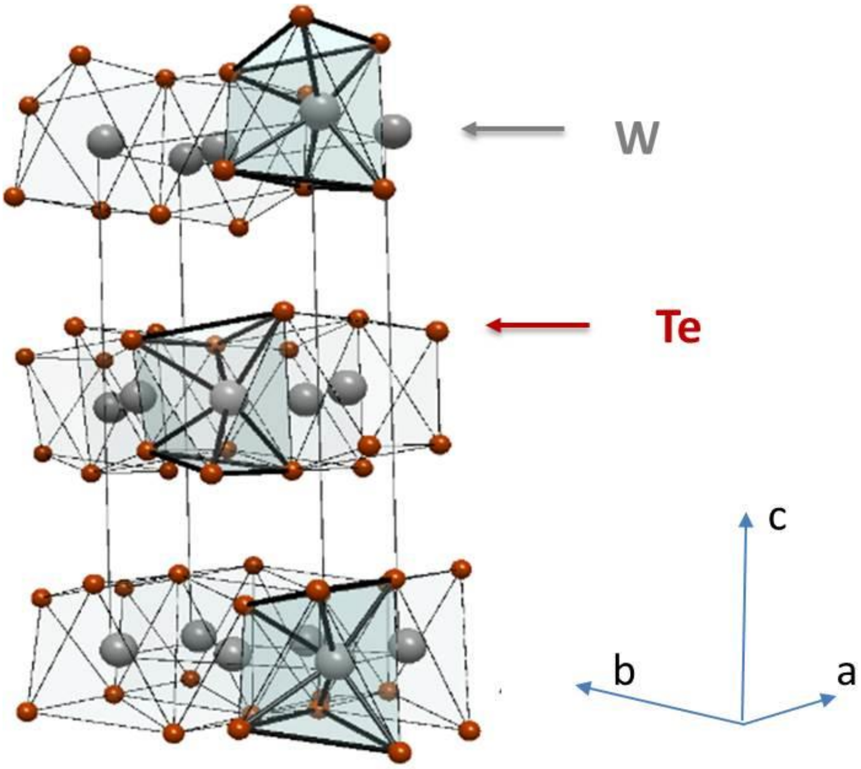

Supplementary Figure 1. **Crystal structure of WTe_2.**

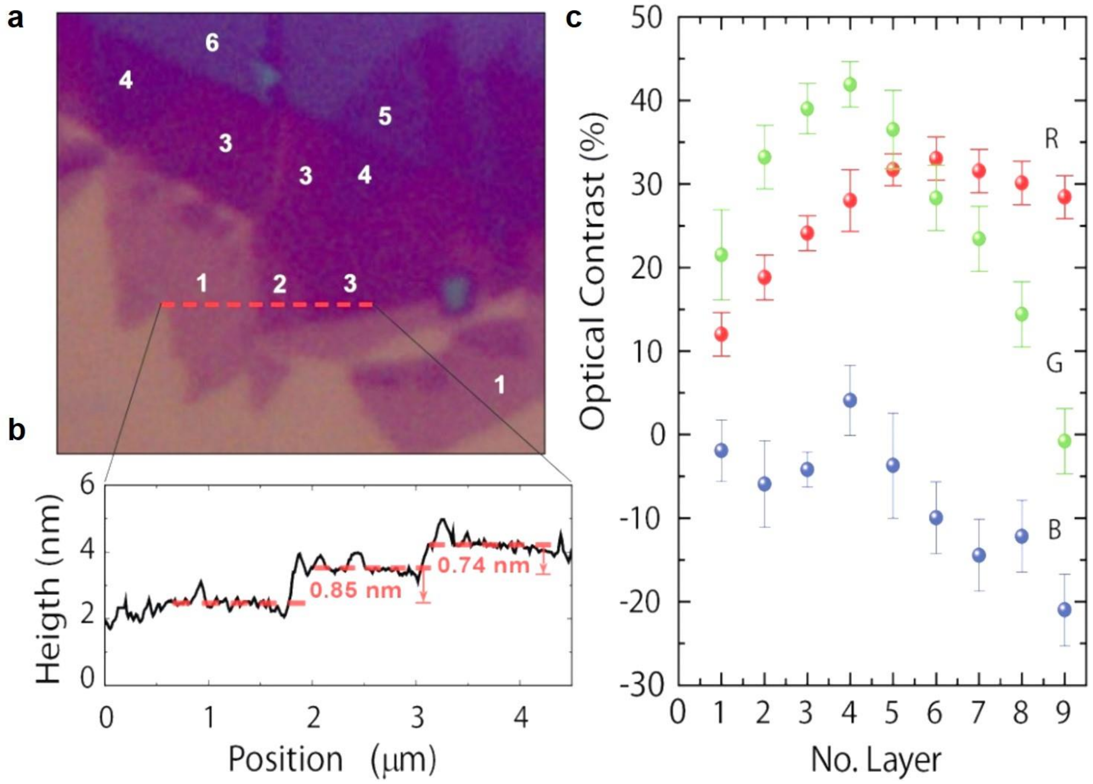

Supplementary Figure 2. **Atomic force microscopy and relative optical contrast of few-layer WTe_2 flakes. (a)** Optical microscope image of an exfoliated WTe_2 flake containing layers of different thickness (from 1 to 6 monolayers). The red dashed line indicates the position at which the atomic force microscopy profile shown in **(b)** was recorded; in the profile, steps corresponding to the expected height of individual WTe_2 monolayers are clearly apparent. The attribution of the absolute number of monolayers also requires optical contrast measurements, which are shown in **(c)**, and the analysis of Raman spectroscopy. The error bars in c are the standard deviation obtained from the analysis of all the flakes with the same thickness.

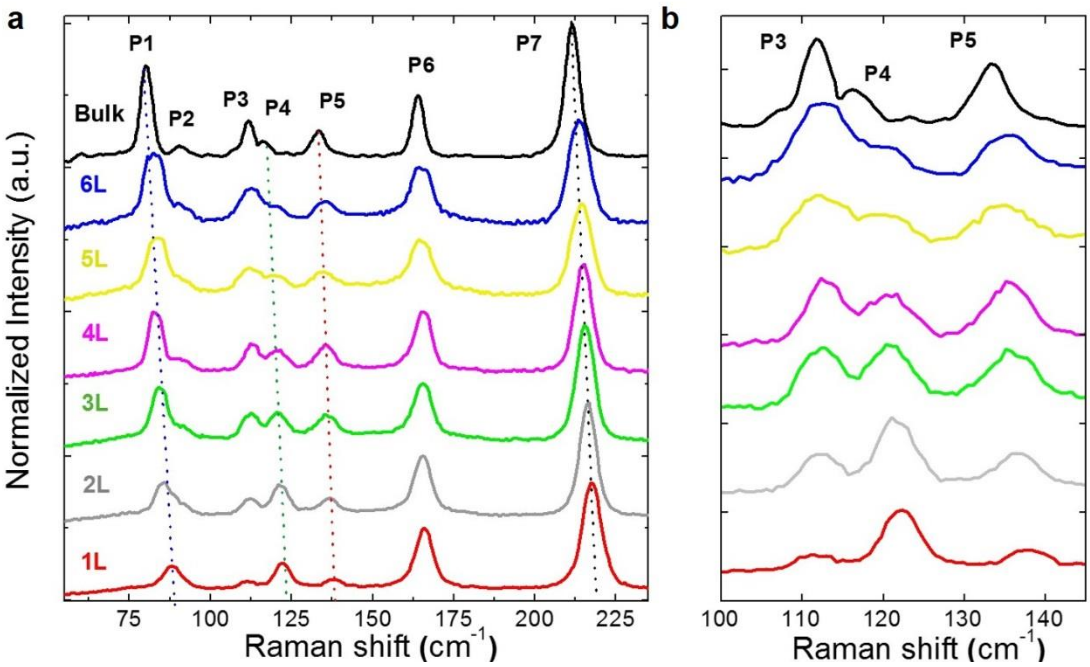

Supplementary Figure 3. Raman spectra of bulk and few-layer WTe_2. (a) Seven peaks, labelled as P1 – P7, are clearly observed in the Raman spectra in the range 50 to 250 cm^{-1} for all thicknesses except for monolayers (peak P2 is missing). The dotted lines are guides to the eye that put in evidence the shift of the peak positions. The range between 100 and 150 cm^{-1}, which shows the evolution of peaks P3 – P5, is enlarged in panel (**b).** All spectra have been offset vertically for clarity.

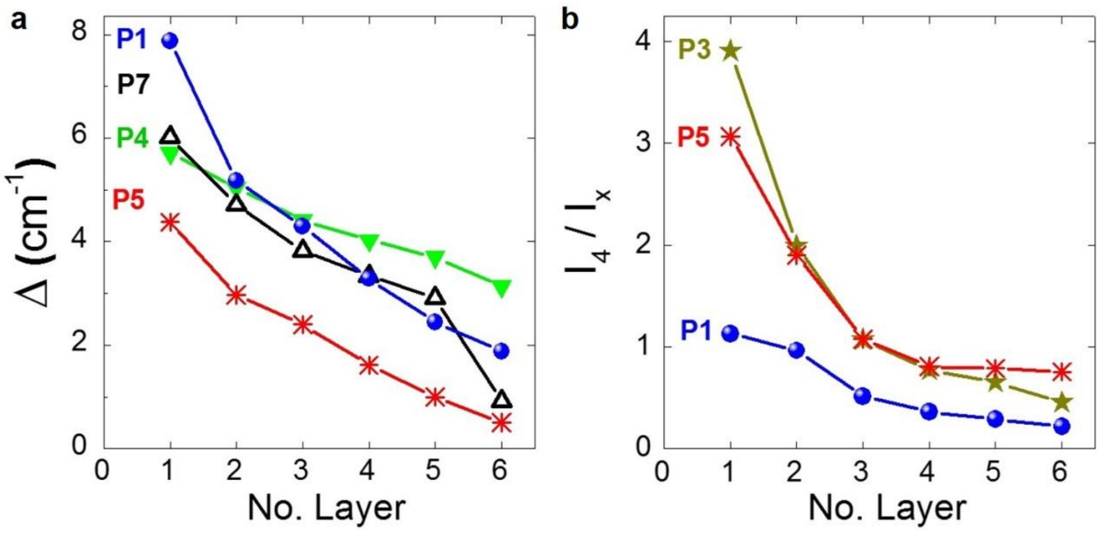

Supplementary Figure 4. Evolution of the position and intensity of selected Raman peaks; P1, P3-P5. (a) Relative Raman shift Δ of peaks P1, P4, P5 and P7 with respect to their bulk values. **(b)** Evolution (as a function of number of layers) of the intensity of peak P4 relative to the intensities of peaks P1, P3 and P5.

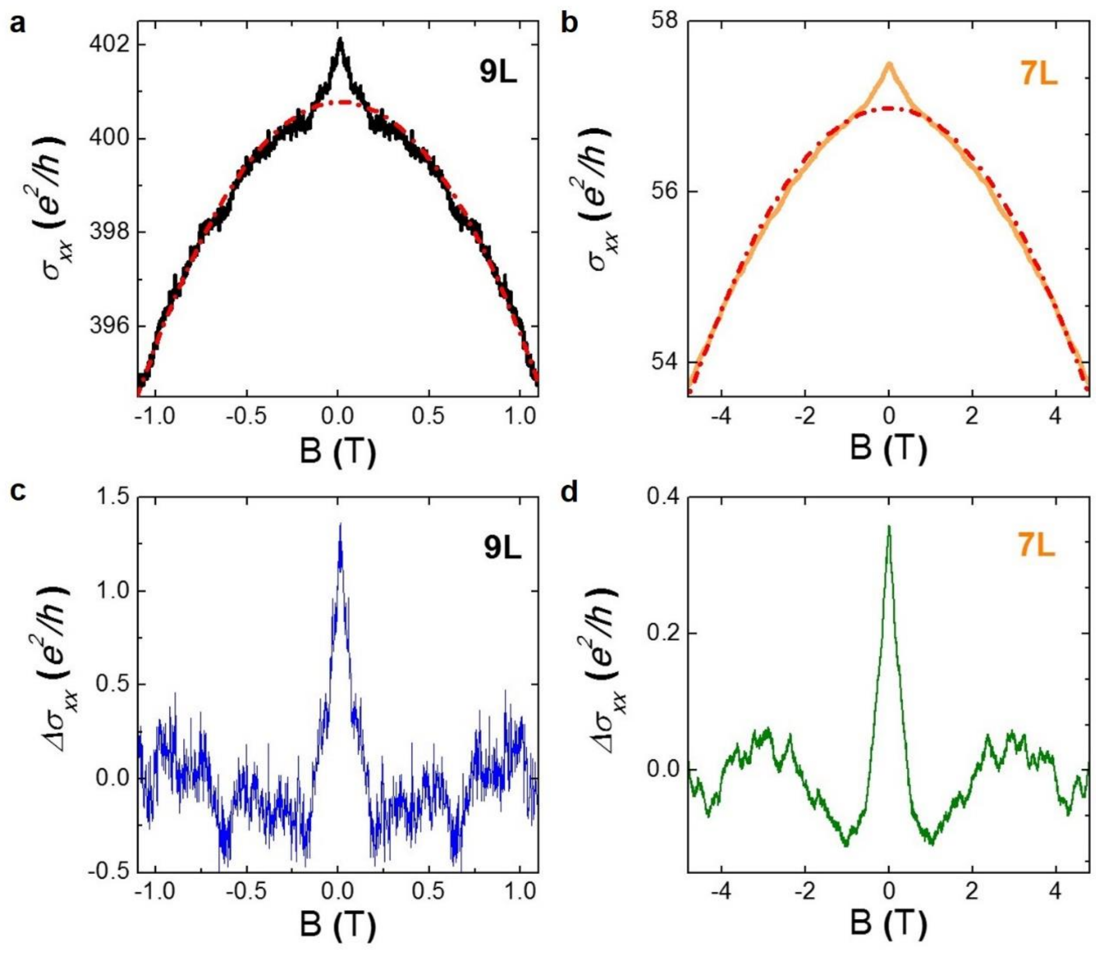

Supplementary Figure 5. Classical and quantum contributions to the magnetoconductance of thin WTe_2 flakes. (a-b) Measured magnetoconductivity (solid line) and classical background (dash-dotted line) obtained from the two-carrier model, of a 9 and a 7 layer flake, respectively. **(c-d)** Quantum corrections to the conductivity (weak antilocalization) obtained by subtracting the quadratic classical background from the measured data shown in (a-b).

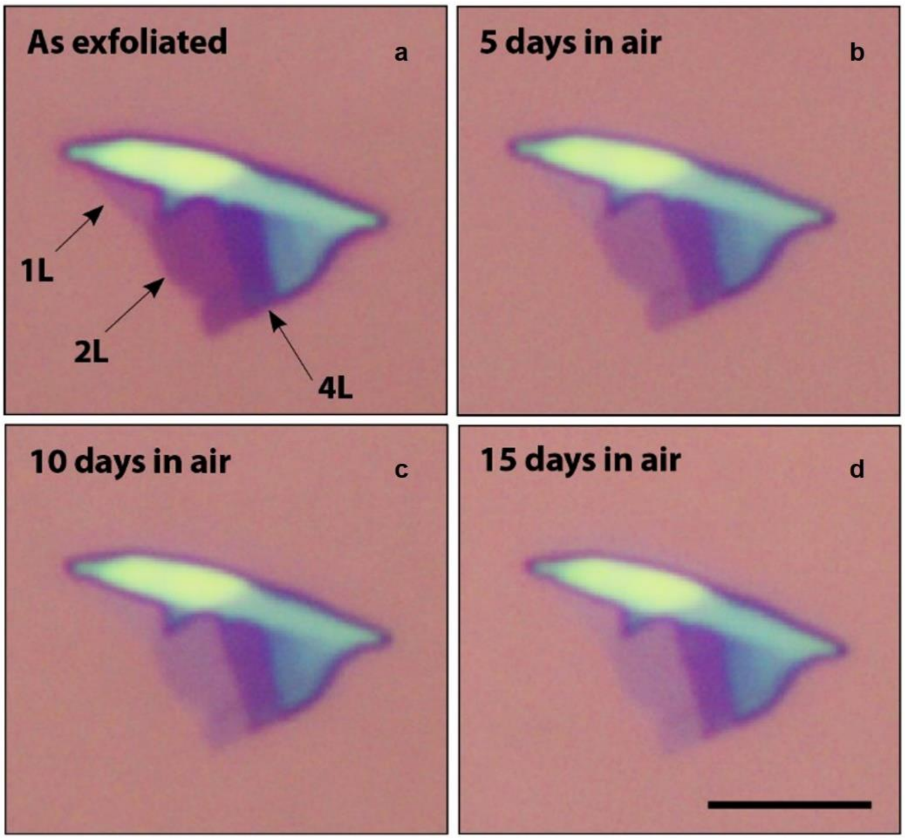

Supplementary Figure 6. Degradation of WTe_2 under ambient conditions. Optical microscope images of a WTe_2 flake **(a)** directly after exfoliation and **(b-d)** after exposing it to ambient conditions for 5, 10 and 15 days, respectively. The arrows in panel (a) point to parts of the flakes corresponding to a monolayer (1L), bilayer (2L) and tetralayer (4L). The scale bar in panel **(d)** is 5 µm.

Supplementary Note 1. Experimental identification of atomically thin flakes

WTe_2 layers of different thickness –all the way down to individual monolayers– were obtained by means of in-air, micro-mechanical cleaving of bulk crystals with an adhesive tape. The exfoliated crystals were subsequently transferred onto substrates consisting of a highly doped silicon wafer covered with a 285 nm layer of thermally grown SiO_2[1], which is known to ensure a good visibility[2,3] of the flakes and can be used as a back gate. More than 30 substrates were systematically inspected under an optical microscope, resulting in the observation of more than 500 atomically thin layers (1 - 9 monolayers thick). An optical picture of one of the many multi-layer flakes found during this work is shown in Supplementary Figure 2a. Regions of different intensity are clearly visible, which correspond to layers of different thickness. As it is often the case for two-dimensional materials, including graphene, atomic force microscopy (AFM) measurements of the step height from the substrate does not allow the actual thickness of the exfoliated crystals to be determined, even though measurements performed across neighboring layers do give a step height corresponding to the expected interlayer spacing of WTe_2 (0.7 nm, as shown in Supplementary Figure 2b). To identify monolayers, we therefore performed a systematic analysis of the optical contrast and Raman spectra of thin WTe_2 exfoliated crystals (for crystals thicker than ~ 10 layers, AFM does allow the thickness to be determined with sufficient accuracy for the purposes of the present work).

Our analysis exploits two facts that are known from previous work on atomically thin layers of other transition metal dichalcogenides (TMDs): both the optical contrast of thin exfoliated flakes relative to the substrate [2–4] and their Raman spectra[4–8] evolve systematically with thickness. For this reason, we conducted a combined analysis of the optical contrast, Raman spectra and AFM height profiles of approximately 50 WTe_2 exfoliated flakes, whose thickness ranged from 1 to 9 monolayers. A first conclusion of this analysis is that layers exhibiting the same intensity under an optical microscope (i.e., having the same thickness) show reproducibly the same optical contrast (Note that it is important to use the same microscope and camera –and to keep the same illumination conditions- to have reproducible values of intensity for any given thickness) and Raman spectra, which makes it possible to univocally assign the layer thickness from a measurement of optical contrast or of Raman spectrum. To assign the thickness correctly, we identified –among the approximately 500 flakes investigated- those parts that have the smallest height relative to the substrate, and found that they systematically correspond to the region of smallest intensity in optical microscope images (even though the actual step height from the substrate is somewhat different in different flakes). Having analyzed a very large number of flakes, we can conclude that these region (exhibiting the smallest intensity values) correspond to individual monolayers. Indeed, since the intensity of monolayers compared to that of the substrate is

rather large, thinner layers should have been easily detected if present. Having identified which parts of the exfoliated flakes correspond to monolayers, the thickness of all other layers (bi, tri, etc.) can be determined by following the height profile in AFM images, since individual crystalline steps are easily detected in this way.

From this analysis we can then assign the measured optical contrast in the R, G, and B channel (The optical contrast is defined as $(I_S - I_F)/I_S$, where I_S and I_F are the intensity of the substrate and the flakes in each of the three RGB channels.) and Raman spectra (see discussion below) univocally to the corresponding thickness of atomically thin WTe_2 flakes. The result of this assignment is summarized in Supplementary Figure 2b (for the RGB contrast), Supplementary Figure 3 (for the Raman spectra), and Supplementary Figure 4 (for the position and intensity of different Raman peaks that are particularly useful to identify the layer thickness).

The Raman spectra of atomically thin WTe_2 crystals, shown in Supplementary Figure 3, were measured at room temperature, with a laser wavelength of 514 nm and a power of 0.7 mW. We also measured the spectrum of a bulk crystal, and confined the detailed analysis to flakes that are six layers or thinner, since the spectrum of thicker flakes is too close to that of bulk crystals to allow the unambiguous determination of the layer thickness. With the exception of monolayers, the Raman spectra of all flakes, exhibit seven distinctive sharp peaks in the range from 50 to 250 cm^{-1}, whose position is close to that of the peaks measured in bulk crystals: P1 = 80.2, P2 = 90.3, P4 = 117.3, P5 = 132.8, P6 = 164.06 and P7 = 211.6 cm^{-1}. Peak P2 is missing in monolayers, which exhibit only six peaks (see Supplementary Figure 3), a feature that facilitates their identification (we note that this conclusion could not be made in previous Raman studies of few-layer WTe_2[9,10], in which measurements were confined to the range between 100 and 250 cm^{-1}). According to a previous theoretical and experimental study of the Raman spectra in WTe_2[11] these peaks originate from either A_1 (P1, P4 - P7) or A_2 (P2 and P3) phonon modes. As for the identification of the layer thickness, the evolution of peaks P1, P3, P4 and P5 is most helpful, as both the position and relative intensities show the largest changes. The analysis of these peaks is summarized in Supplementary Figure 4a (peak positions) and Supplementary Figure 4b (relative intensity).

Supplementary Note 2. Classical and quantum contributions to the magnetoconductance of atomically thin WTe_2 flakes.

Decreasing the thickness of WTe_2 flakes below 10 layers makes the quantum correction to the conductivity due to weak antilocalization (WAL, as discussed in the main text) experimentally visible. In flakes that are 9 and 7 layer thick it is possible to separate unambiguously the contribution to the conductivity of classical origin, from the quantum correction due to WAL. Supplementary Figure 5 shows separately the classical and quantum

contributions to the magnetoconductance: the classical contribution corresponds to the parabolic dashed-dotted line fit to the data; the quantum correction is obtained by subtracting the classical contribution from the total measured conductivity. Although a complete analysis of the WAL contribution would require a precise knowledge of the form of spin-orbit interaction, we find –as expected- that the magnitude of the quantum correction to the conductivity is ~ e^2/h (see also the data for the 4 layer flake in Fig. 4a of the main text).

Supplementary Note 3. Environmental stability of WTe_2 atomically thin flakes

The degradation of atomically thin WTe_2 flakes under ambient conditions is clearly visible in the sequence of optical microscope images shown in Supplementary Figure 6. The images show that regions thinner than 4 layers undergo a change in color reminiscent of the degradation seen in thin flakes of other materials, such as black phosphorous[12] or $NbSe_2$[13]. However, unlike black phosphorous, in which the degradation of flakes several layers thick (including the formation of bubbles or droplets on the surface) can be seen already after one hour of exposure to air[12], thicker regions of WTe_2 (tenths of nanometers) do not show clear sign of degradation even after 15 days under ambient conditions. These observations are consistent with the strong decrease in mobility and the occurrence of a metal-insulator transition when the thickness of WTe_2 is reduced below 4 layers (as discussed in the main text).

Supplementary Note 4. Growth of WTe_2 crystals

Single crystals of WTe_2 were grown by means of chemical vapor transport using WCl_6 as a transport agent. As a first step, pure elements W and Te were mixed together with the transport agent WCl_6 in a stoichiometric cation ratio, according to the reaction equation:

$$\left(\frac{n-1}{n}\right)W + \frac{1}{n}\,WCl_6 + 2X \rightarrow WTe_2 + \frac{3}{n}\,Cl_2 \quad \text{with } n = 15$$

The total material weight used in each growth was about 0.2 - 0.3 g. The mixture was prepared and weighted in a glove box and sealed (under vacuum, $p \sim 5 \times 10^{-6}$ mbar) in a quartz ampule with an internal diameter of 8 mm and a length of 120 mm. The sealed quartz reactor was heated up in a two zone furnace in the presence of a thermal gradient $dT/dx \approx 5$ – 10 °C/cm, with the hot end at T_{hot} = 890°C and the cold end at T_{cold} = 790°C. After keeping these conditions for four days, the furnace was switched off and cooled down to room

temperature. As a result of the process, the whole precursor load moved from the hot side to the cold side of the quartz ampule, where crystals could be found, either isolated or aggregated together. After removing them from the quartz wall, these crystals were either cleaved or cut, and subsequently characterized by X-ray diffraction (XRD) and SEM-EDX analysis. X-Ray diffraction and structure refinement proved that all the crystals crystallized in the orthorhombic $P\ m\ n\ 2_1$ space group[14]. This unique polymorph (see Supplementary Figure 1) of WTe_2 derives from distortion of the 1T octahedral and is commonly referred to as the "1T' "structure. Within the accuracy of the EDX probe, the atomic ratio W:Te was found to be uniformly equal to 1:2 throughout the crystal inspected, in agreement with the XRD results.

Supplementary References

1. Novoselov, K. S. *et al.* Two-dimensional atomic crystals. *Proc. Natl. Acad. Sci. U. S. A.* **102,** 10451–3 (2005).

2. Benameur, M. M. *et al.* Visibility of dichalcogenide nanolayers. *Nanotechnology* **22,** 125706 (2011).

3. Castellanos-Gomez, a., Agraït, N. & Rubio-Bollinger, G. Optical identification of atomically thin dichalcogenide crystals. *Appl. Phys. Lett.* **96,** 213116 (2010).

4. Lezama, I. G. *et al.* Indirect-to-Direct Band Gap Crossover in Few-Layer $MoTe_2$. *Nano Lett.* **15,** 2336–2342 (2015).

5. Lee, C. *et al.* Anomalous lattice vibrations of single- and few-layer MoS_2. *ACS Nano* **4,** 2695–2700 (2010).

6. Berkdemir, A. *et al.* Identification of individual and few layers of WS_2 using Raman spectroscopy. *Sci. Rep.* **3,** 1755 (2013).

7. Li, S. *et al.* Quantitative Raman spectrum and reliable thickness identification for atomic layers on insulating substrates. *ACS Nano* **6,** 7381–7388 (2012).

8. Yamamoto, M. *et al.* Strong Enhancement of Raman Scattering from a Bulk-Inactive Vibrational Mode in Few-Layer $MoTe_2$. *ACS Nano* **4,** 3895 – 3903 (2014).

9. Jiang, Y. C. & Gao, J. Raman fingerprint of semi-metal WTe_2 from bulk to monolayer. Preprint at http:// http://arxiv.org/abs/1501.04898 (2015).

10. Jana, M. K. *et al.* A combined experimental and theoretical study of the electronic and vibrational properties of bulk and few-layer Td-WTe_2. Preprint at http:// http://arxiv.org/abs/1502.04171 (2015).

11. Kong, W.-D. *et al.* Raman scattering investigation of large positive magnetoresistance material WTe_2. *Appl. Phys. Lett.* **106,** 081906 (2015).

12. Castellanos-Gomez, A. *et al.* Isolation and characterization of few-layer black phosphorus. *2D Mater.* **1,** 025001 (2014).

13. Geim, a K. & Grigorieva, I. V. Van der Waals heterostructures. *Nature* **499,** 419–425 (2013).

14. Mentzen, B. F. & Sienko, M. J. Preparation and x-ray study of mixed-anion tungsten dichalcogenides. *Inorg. Chem.* **15,** 2198–2202 (1976).
